# Population‐Based Versus Hospital‐Based Data in Amyotrophic Lateral Sclerosis—A Factor to Consider?

**DOI:** 10.1111/ene.70137

**Published:** 2025-04-04

**Authors:** Johannes Dorst, Jens Dreyhaupt, Deborah Wernecke, Ulrike Weiland, Özlem Parlak, Maximilian Wiesenfarth, Zeynep Elmas, Christine Herrmann, Hansjörg Bäzner, Axel Boertlein, Silke Dempewolf, Christian Foerch, Martin Hecht, Andreas Kohler, Christian Opherk, Katharina Althaus, Monika Clauer‐Bredt, Alfred Lindner, Wolfgang Ruf, David Brenner, Simon Witzel, Raphael S. Peter, Joachim Schuster, Albert C. Ludolph, Angela Rosenbohm, Gabriele Nagel

**Affiliations:** ^1^ Department of Neurology University of Ulm Ulm Germany; ^2^ German Center for Neurodegenerative Diseases (DZNE) Ulm Germany; ^3^ Institute of Epidemiology and Medical Biometry University of Ulm Ulm Germany; ^4^ Department of Neurology Katharinenhospital Stuttgart Stuttgart Germany; ^5^ Department of Neurology RKH Klinikum Ludwigsburg Ludwigsburg Germany; ^6^ Department of Neurology Klinikum Kaufbeuren Kaufbeuren Germany; ^7^ Department of Neurology Klinikum Am Gesundbrunnen Heilbronn Heilbronn Germany; ^8^ Department of Neurology Christophsbad Goeppingen Goeppingen Germany; ^9^ Department of Neurology Marienhospital Stuttgart Stuttgart Germany

**Keywords:** amyotrophic lateral sclerosis, hospital‐based data, population‐based data, prognosis, selection bias

## Abstract

**Background:**

Over the past years, some studies in amyotrophic lateral sclerosis (ALS) have provided heterogeneous findings regarding demographic and clinical data as well as the impact of various prognostic factors. It is well known that these inconsistencies might be caused by a selection bias in hospital‐based data sets. In this study, we sought to further characterize this selection bias.

**Methods:**

We compared hospital‐based data from the ALS center at Ulm University (UC; *n* = 3833; 1997–2021) with the population‐based ALS registry Swabia (SR; *n* = 852; 2010–2020).

**Results:**

Patients from UC were younger (age of onset 60.9 [IQR 52.4–68.9] vs. 65.0 [57.0–72.7]), had a higher share of males (60.5% vs. 56.3%), a longer diagnostic delay (10.5 [IQR 6.4–18.4] months vs. 6.9 [IQR 3.4–12.1] months), a higher prevalence of the “definite” category according to El Escorial diagnostic criteria (60.9% vs. 11.2%), a higher share of familial cases (12.9% vs. 6.3%), a slower progression rate (points of ALS functional rating scale revised lost per month −0.54 [IQR −1.02 to −0.28] vs. −0.79 [IQR −1.47 to −0.43]), and (among all deceased patients) a higher share of percutaneous endoscopic gastrostomy (26.7% vs. 17.7%) and non‐invasive ventilation (34.3% vs. 25.3%).

**Conclusions:**

The observed differences likely indicate a selection bias in hospital‐based data, which may be attributed, among others, to the willingness to travel large distances to a specialized center, the desire to participate in clinical studies, and the attitude toward life‐prolonging measures. These differences must be considered when interpreting and generalizing study results from hospital‐based populations.

## 1 Introduction

Amyotrophic Lateral Sclerosis (ALS) is a fatal neurodegenerative disease characterized by the loss of upper (UMN) and lower motor neurons (LMN) and leading to death usually caused by respiratory failure after a mean disease duration of only 2–4 years [1]. It has been shown that pTDP‐43, a pathologically misfolded protein, continuously spreads through the brain and spinal cord [2]. However, so far, this finding has not been translated into an effective therapy [3].

Since therapeutic options are limited, it is important to know about factors that modify prognosis, stratify for these factors in clinical studies, and identify additional therapeutic targets. Among others, younger age of onset, initial spinal manifestation, as well as better respiratory and neuropsychological status have been established as factors positively influencing prognosis [3, 4], while the prognostic influence of gender is quite unclear. ALS patients also feature a variety of metabolic alterations that seem to interact with the course of the disease. Lower body mass index (BMI) and loss of body weight have repeatedly been described as factors negatively affecting survival [5, 6], and targeting these factors via high‐caloric nutritional interventions has yielded promising results [7, 8], although the highest level of evidence has still not been provided. More controversially, alterations of lipid [9, 10, 11] and carbohydrate metabolism [12, 13] have also been associated with prognosis. Percutaneous endoscopic gastrostomy (PEG) and non‐invasive ventilation (NIV) constitute important symptomatic treatment options that mainly aim at improving quality of life. While the latter has repeatedly been reported to also prolong survival [14, 15], the impact of PEG on survival is still unclear.

Some of these prognostic factors are discussed quite controversially in literature, based on seemingly inconsistent or even contradictory results. One example is the effect of sex, as female gender (possibly confounded by a higher share of bulbar onsets) has been described to be associated with shorter [16, 17] as well as longer survival [18, 19], while most studies indicated no association [20]. With regard to comorbidities, type II diabetes has been found to be associated with later ALS onset [12, 21] and decreased risk of ALS [13, 22], suggesting a protective effect, but, on the other hand, was not associated with prolonged survival [13]. Likewise, dyslipidemia has been repeatedly described as a positive prognostic factor [9, 10], while other studies have not found an independent effect [3, 23]. Related to the same issue, the prescription of statins has also been discussed controversially, as some studies suggested a negative effect [24], while subsequent studies could not reproduce this [25]. The effect of various therapeutic measures on survival is also quite unclear. For example, some older studies suggested a beneficial effect of PEG [26], while a recent meta‐analysis including 12 studies with overall heterogeneous findings indicated no effect [20]. Even treatments that are generally believed to prolong life, such as NIV, show wide ranges with regard to the extent of survival benefit [15, 27, 28].

The overt heterogeneity of these and other ALS‐related findings may be explained by multiple factors, including genetic and environmental influences and varying methodologies (e.g., regarding adjustment for potential confounding factors) [29]. However, another factor to consider is related to the source of data, that is, whether data were derived from specialized ALS centers, population‐based registries, or other sources with high coverage [30, 31, 32]. While hospital‐based data are prone to selection bias, possible pitfalls in population‐based approaches include changing demographics and the use of prevalent rather than incident cases [33, 34]. A study based on data from the EURALS consortium revealed that patients referred to the ALS center were younger, less likely to present with a bulbar onset, had a higher proportion of familial cases, and a longer survival compared to population‐based cohorts from the same geographic area [31].

Therefore, in this study, we aimed at describing demographic and clinical differences between hospital‐ and population‐based cohorts, as well as their prognostic impact. To that end, we compared a large database consisting of 3833 patients from a specialized ALS tertiary center (Ulm University Hospital, Ulm Center, UC) with 852 patients from the population‐based ALS registry Swabia (Swabia Registry, SR), which was established in 2010 with the aim to collect data of all ALS cases diagnosed in a geographically defined region [35]. As both cohorts are in the same geographic region, there is an overlap of 669 patients who are part of both datasets. Importantly, this overlap is intended based on the scientific purpose of this study.

## 2 Methods

This study is a comparison between a large hospital‐based database from the specialized ALS center at Ulm University (Ulm Center; UC) and a population‐based registry from Swabia, a defined target area around Ulm (Swabia Registry; SR). Ethical approval was obtained from the ethical committees of Ulm University (reference # 11/10) and the regional medical associations (Landesärztekammer Baden‐Württemberg reference # B‐F‐2010‐062 and Landesärztekammer Bayern reference # 7/11300). The study was undertaken with the understanding and written consent of each subject, and the study conforms with the World Medical Association Declaration of Helsinki.

### 2.1 Hospital‐Based Database From Ulm University

The UC hospital database is based on data collected between 1997 and 2021 in the specialized ALS center of the Neurological Department of Ulm University. In this dataset (*n* = 3833), all patients with possible, probable, or definite ALS according to revised El Escorial criteria [36] who were treated either in hospital or in the outpatient clinic between 1997 and 2021 were included. To obtain a more homogenous population, patients with pure upper (UMN) or lower motor neuron (LMN) subforms, such as Primary Lateral Sclerosis (PLS) and Progressive Muscular Atrophy (PMA) were excluded, and only patients featuring a typical ALS phenotype with simultaneous affection of UMN and LMN were considered, including patients with Flail‐Arm‐(FAS)/Flail‐Leg‐Syndrome (FLS), ALS with frontotemporal dementia (ALS‐FTD), and genetic forms.

In general, this cohort is characterized by the following groups of patients:

1. Patients who were referred to the center by external neurologists for a second opinion,
2. Patients who sought to participate in a clinical study; these patients were sent from various regions of Germany, and
3. Patients located in the geographic region around Ulm who were seeking regular treatment; these patients constitute the overlap with SR (see below).

Regular treatment included outpatient visits in 3–6‐monthly intervals, while hospitalization usually occurred either for extensive diagnostics (second opinion, subgroup 1), for crisis intervention (e.g., aspiration pneumonia), or to establish PEG, NIV, or invasive ventilation (IV) (subgroup 3).

For each patient, the following information was systematically collected in a standardized manner: date of birth, sex, diagnostic revised criteria (El Escorial [36]), sporadic or familial form, date of onset (defined as occurrence of first paresis), spinal or bulbar onset, proximal or distal onset, right or left onset, clinical phenotype, ALSFRS‐R, BMI, forced vital capacity (FVC), date of PEG, date of NIV, and date of tracheostomy.

### 2.2 ALS Registry Swabia

The SR is a population‐based clinical‐epidemiological registry in a defined geographic region in the South‐West of Germany (details see [35]), including the city of Ulm from which the hospital‐based data were obtained (see above). The catchment area has approximately 8.4 million inhabitants. The aim of the registry is to collect data on all newly diagnosed ALS patients who had their residence in Swabia. Consequently, the registry provides estimates of epidemiological variables, such as incidence, and describes the natural history of ALS, including survival status. It further allows for the investigation of risk factors for ALS by means of a registry‐based case‐control study, which is, however, not part of this analysis.

Beginning on October 01, 2010, all newly diagnosed ALS patients (until 2020, *n* = 1739) were registered prospectively. ALS patients were defined by the diagnosis of possible, probable, or definite ALS according to the revised El Escorial criteria [35]. To synchronize the datasets for this analysis, all patients who did not agree to be visited by a study nurse to obtain baseline and follow‐up data were excluded (*n* = 568). In addition, patients with the El Escorial category of “suspected” ALS at baseline (*n* = 319) were excluded to match the inclusion/exclusion criteria for the hospital‐based database. The remaining dataset consisted of *n* = 852 patients.

Amyotrophic lateral sclerosis patients were actively followed up on a yearly basis by performing a standardized interview. For survival status, the registration offices were contacted annually, and, if a patient was deceased, the date of death was received (last systematic mortality update in December 2020). Collected demographic and clinical parameters match those described above for the hospital‐based dataset, except for FVC, which was not available for the SR.

### 2.3 Statistics

Continuous data are given as median and interquartile range (IQR). Categorical data are presented as frequencies and percentages. Pre‐baseline progression rate (measured as points of ALSFRS‐R lost per month) was calculated by the formula: (48—ALSFRS‐R at first visit)/months between onset (first paresis) and first visit. Follow‐up progression rate was calculated by the formula: (ALSFRS‐R at first visit—ALSFRS‐R at last visit)/months between first and last visit.

Survival time was defined by the time between the onset of first paresis and death/last systematic mortality follow‐up. For survival analysis, the log‐rank test was used, and Kaplan–Meier curves were presented. To estimate the effect of prognostic factors, we used univariate as well as mutually adjusted multivariable Hazard Ratios (HRs) from the Cox proportional hazard regression model, including a 95% confidence interval (CI). Model entry for observed survival time was the time of the first visit (baseline visit) to adjust for immortal time bias [37]. To analyze the effect of NIV, invasive ventilation via tracheostomy (TIV), and PEG on survival, only deceased patients were considered, because their status regarding NIV, TIV, or PEG was known.

The level of significance was set at *p* = 0.05 (two‐sided). Because of the explorative nature of this study, all results from the statistical tests must be interpreted as hypothesis generating only and not as confirmatory. The analyses were performed using SAS 9.4 (The SAS Institute, Cary, NC, USA).

## 3 Results

For UC and SR, respectively, 3833 and 852 patients fulfilled the inclusion/exclusion criteria as described above and were analyzed. A total of 669 patients were part of both cohorts.

### 3.1 Demographic and Clinical Characteristics

**FIGURE 1:**
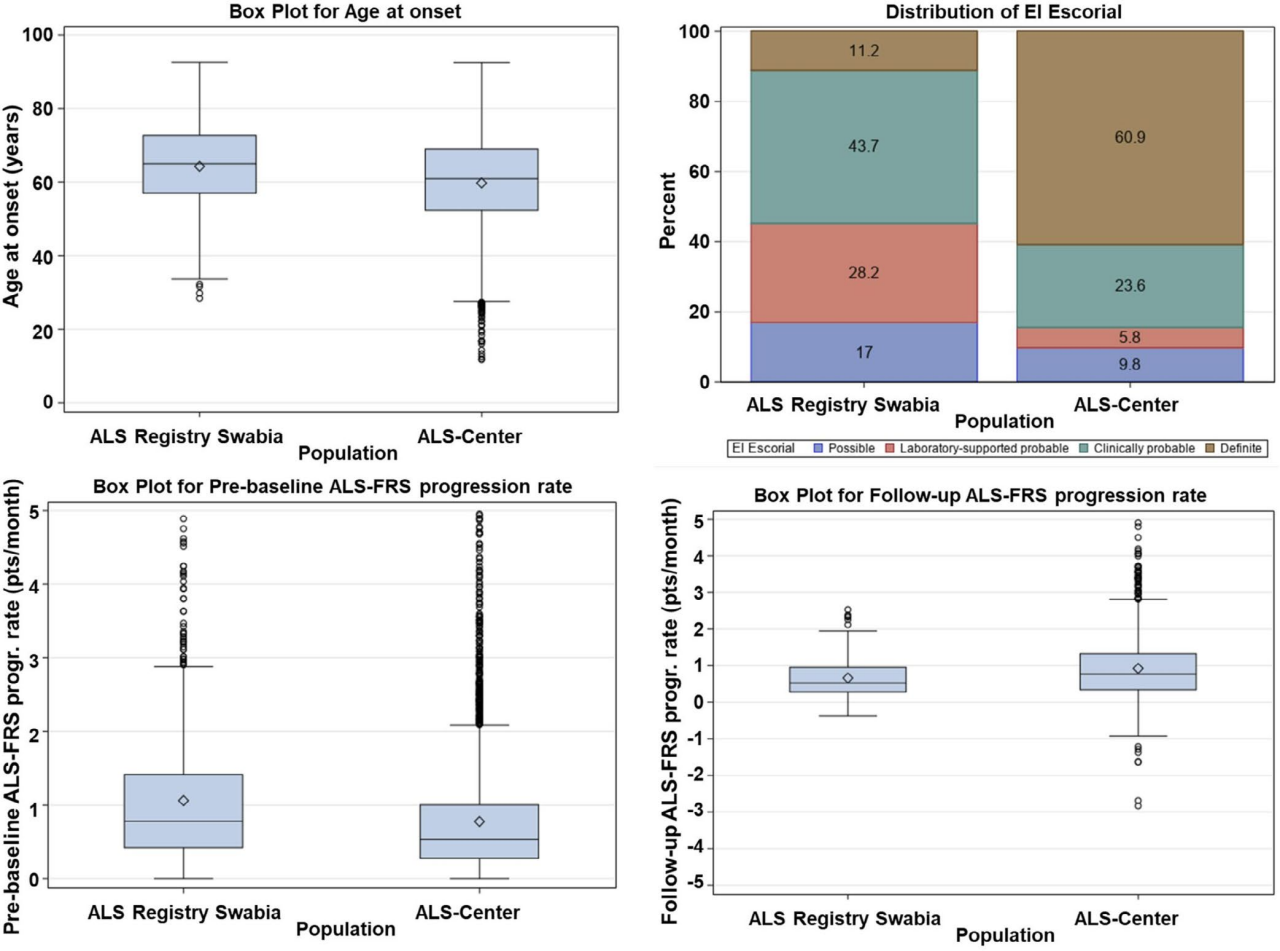
Demographic and clinical characteristics. Comparison of demographic and clinical characteristics between the ALS Registry Swabia (left, *n* = 852) and the Ulm ALS center (right, *n* = 3833). Upper left: Age of onset; upper right: Distribution according to El Escorial criteria diagnostic categories; lower left: Pre‐baseline ALSFRS‐R progression rate (points of ALSFRS‐R lost per month); lower right: Follow‐up ALSFRS‐R progression rate (points of ALSFRS‐R lost per month). ALSFRS‐R, amyotrophic lateral sclerosis functional rating scale revised.

Demographic and clinical characteristics are displayed in Table 1 and Figure 1. Compared to the population‐based SR data, patients from UC were younger (age of onset 60.9 [IQR 52.4–68.9] vs. 65.0 [57.0–72.7]), had a higher share of males (60.5% vs. 56.3%), a longer diagnostic delay (10.5 [IQR 6.4–18.4] months vs. 6.9 [IQR 3.4–12.1] months), a higher share of the “definite” category according to El Escorial diagnostic criteria (60.9% vs. 11.2%), a higher share of familial cases (12.9% vs. 6.3%), a slower pre‐baseline ALSFRS‐progression rate (ALSFRS‐R points lost per month −0.54 [IQR −1.02 to −0.28] vs. −0.79 [IQR −0.43 to −1.47]), a faster follow‐up progression rate (ALSFRS‐R points lost per month −0.78 [IQR −1.36 to 0.35] vs. −0.53 [−0.96 to 0.28]), and (among all deceased patients) a higher share of percutaneous endoscopic gastrostomy (26.7% vs. 17.7%) and non‐invasive ventilation (34.3% vs. 25.3%). The share of patients with bulbar onset was slightly higher (33.9% vs. 31.2%). We did not find any differences regarding ALSFRS‐R score, BMI, and share of patients with tracheotomy. The lower share of deceased patients (57.8% vs. 74.3%) is most likely explained by the higher lost‐to‐follow‐up rate and shorter mean follow‐up time in the hospital cohort (9.2 [IQR 0.0–21.7] vs. 16.2 [IQR 8.4–31.0] months).

**TABLE 1:** Demographic and clinical characteristics.

Variable		ALS‐Center Ulm (UC) (*n* = 3833)		ALS Registry Swabia (SR) (*n* = 852)
Observation time		02/1997–01/2021		10/2010–12/2020
	***N***		***N***	
**Age at diagnosis** (years)	1793		852	
Median (Q1, Q2)		62.8 (54.2, 70.9)		65.6 (57.6, 73.8)
Mean (SD)		61.8 (11.8)		65.1 (11.2)
**Age at onset** (years)	3796		852	
Median (Q1, Q2)		60.9 (52.4, 68.9)		65.0 (57.0, 72.7)
Mean (SD)		59.7 (12.2)		64.3 (11.2)
**Sex**, *N* (%)	3833		852	
Female		1513 (39.5)		372 (43.7)
Male		2320 (60.5)		480 (56.3)
**Diagnostic delay** (months)	1781		852	
Median (Q1, Q2)		10.5 (6.4, 18.4)		6.9 (3.4, 12.1)
Mean (SD)		15.6 (18.8)		9.8 (10.1)
**El Escorial**, *N* (%)	3736		852	
Definite		2275 (60.9)		95 (11.2)
Clinically probable		880 (23.6)		372 (43.7)
Laboratory‐supported probable		216 (5.8)		240 (28.2)
Possible		365 (0.8)		145 (17.0)
**Family history** (anamnesis), *N* (%)	3833		852	
Familial		494 (12.9)		54 (6.3)
Sporadic		3324 (86.7)		798 (93.7)
Unknown		15 (0.4)		—
**Phenotype** (clinical exam), *N* (%)	1875		810	
UMN predominant		90 (4.8)		32 (4.0)
LMN predominant		336 (17.9)		11 (1.4)
UMN/LMN about equally affected		1273 (67.9)		757 (93.5)
FAS/FLS		136 (7.3)		10 (1.2)
ALS‐FTD		40 (2.1)		16 (1.8)
**Onset** (anamnesis), *N* (%)	2964		808	
Bulbar		1006 (33.9)		252 (31.2)
Spinal		1958 (66.1)		556 (68.8)
	1481			
Distal		424 (28.6)	591	67 (11.3)
Proximal		125 (8.4)		9 (1.5)
Simultaneous or not recalled		932 (62.9)		515 (87.1)
	1512		575	
Right		646 (42.7)		208 (36.2)
Left		658 (43.5)		206 (35.8)
Simultaneous or not recalled		208 (13.8)		161 (28.0)
**ALS‐FRS‐R at baseline**	3341		776	
Median (Q1, Q3)		40.0 (35.0, 44.0)		38.0 (33.0, 42.0)
Mean (SD)		38.0 (7.8)		36.8 (7.2)
**Pre‐baseline ALS‐FRS‐R progression rate**	3321		776	
(points of ALFRS‐R lost per month)				
Median (Q1, Q2)		−0.54 (−1.02, −0.28)		−0.79 (−1.47, −0.43)
Mean (SD)		−0.87 (1.88)		−1.15 (1.20)
**Follow‐up ALS‐FRS‐R progression Rate**	1419		273	
(points of ALSFRS‐R lost per month)				
Median (Q1, Q2)		−0.78 (−1.36, 0.35)		−0.53 (−0.96, −0.28)
Mean (SD)		−1.02 (1.16)		−0.67 (0.54)
**BMI**	2734		771	
Median (Q1, Q3)		24.2 (21.8, 26.8)		24.5 (21.8, 27.2)
Mean (SD)		24.5 (4.2)		24.8 (4.6)
**FVC** (%)	2896			No data
Median (Q1, Q3)		80.0 (63.0, 94.0)		
Mean (SD)		77.9 (23.4)		
**PEG**				
Deceased patients only	2214		633	
Yes		592 (26.7)		112 (17.7)
No		1618 (73.1)		519 (82.0)
Missing		4 (0.2)		2 (0.3)
All patients	3833		852	
Yes		768 (20.0)		24 (11.0)
No		3028 (79.0)		136 (16.0)
Missing		37 (1.0)		714 (83.8)
**NIV**	2214		633	
Deceased patients only				
Yes		759 (34.3)		160 (25.3)
No		1451 (65.5)		472 (74.6)
Missing		4 (0.2)		1 (0.2)
All patients	3833			
Yes		960 (25.0)		215 (25.2)
No		2836 (74.0)		636 (74.7)
Missing		37 (1.0)		1 (0.1)
			852	
**Tracheostomy**	3833		852	
Yes		99 (2.6)		23 (2.7)
No		3697 (96.5)		827 (97.1)
Missing		37 (1.0)		2 (0.2)
**Died** *N* (%)	3833	2214 (57.8)	852	633 (74.3)
**Follow‐up time**	3331		852	
(months; baseline to death/last follow‐up)				
Median (Q1, Q3)		9.2 (0.0, 21.7)		16.2 (8.4, 31.0)
Mean (SD)		16.1 (22.2)		24.1 (22.7)

*Note:* Demographic and Clinical Characteristics of the whole study population (*n* = 3833).

Abbreviations: ALSFRS‐*R*, amyotrophic lateral sclerosis functional rating scale revised; ALS‐FTD, amyotrophic lateral sclerosis with frontotemporal dementia; BMI, Body Mass Index; FAS, Flail Arm Syndrome; FLS, Flail Leg Syndrome; FVC, forced vital capacity; LMN, lower motor neuron; NIV, non‐invasive ventilation; PEG, percutaneous endoscopic gastrostomy; UMN, upper motor neuron.

### 3.2 Prognostic Factors

We found via univariate Cox regression analysis (Table 2) that, in both datasets, younger age of onset (UC: HR 0.59 [95% CI: 0.54–0.65]; *p* < 0.001, SR: HR 0.53 [95% CI: 0.45–0.62]; *p* < 0.001), spinal onset (UC: HR 0.63 [95% CI: 0.56–0.70]; *p* < 0.001, SR: HR 0.73 [95% CI: 0.60–0.87]; *p* = 0.001), slower pre‐baseline progression rate (UC: HR 0.45 [95% CI: 0.41–0.49]; *p* < 0.001, SR: HR 0.41 [95% CI: 0.35–0.49]; *p* < 0.001), higher BMI (UC: HR 0.78 [0.70–0.87]; *p* < 0.001, SR: HR 0.82 [95% CI: 0.70–0.96]; *p* = 0.01), and TIV (UC: HR 0.54 [95% CI: 0.41–0.71]; *p* < 0.001, SR: HR 0.34 [95% CI: 0.18–0.66]; *p* = 0.002) were associated with longer survival. Male sex (HR 0.80 [95% CI: 0.68–0.93]; *p* = 0.004) was associated with longer survival only in the SR, but not in the UC cohort, and PEG (HR 0.90 [95% CI: 0.81–1.00]; *p* = 0.04) was associated with longer survival only in the UC, but not in the SR cohort.

**TABLE 2:** Crude hazard ratios for different prognostic factors for survival with ALS.

	ALS Center Ulm (*N*_Deceased_ = 2214, *N*_Survived_ = 1619)			ALS Registry Swabia (*N*_Deceased_ = 633, *N*_Survived_ = 219)		
Exposure	*N*	HR (95% CI)	*p*	*N*	HR (95% CI)	*p*
Age of onset (<median)	2440	**0.59 (0.54, 0.65)**	**< 0.001**	852	**0.53 (0.45, 0.62)**	**< 0.001**
Sex (female vs. male)	2440	1.06 (0.97, 1.16)	0.20	852	**1.26 (1.08, 1.47)**	**0.004**
Spinal onset (vs bulbar)	1862	**0.63 (0.56, 0.70)**	**< 0.001**	808	**0.73 (0.60, 0.87)**	**< 0.001**
Slower pre‐baseline progression rate (< median)	2440	**0.45 (0.41, 0.49)**	**< 0.001**	776	**0.41 (0.35, 0.49)**	**< 0.001**
Positive family history of ALS	2434	0.98 (0.85, 1.13)	0.80	852	1.10 (0.80, 1.50)	0.55
BMI at baseline (> median)	1861	**0.78 (0.70, 0.87)**	**< 0.001**	771	**0.82 (0.70, 0.96)**	**0.01**
**Deceased patients only**						
NIV (ever vs. never)	1875	1.01 (0.92, 1.11)	0.97	632	0.87 (0.72, 1.04)	0.12
TIV (ever vs. never)	1875	**0.54 (0.41, 0.71)**	**< 0.001**	631	**0.34 (0.18, 0.66)**	**0.002**
PEG (ever vs. never)	1875	**0.90 (0.81, 1.00)**	**0.04**	631	0.86 (0.70, 1.06)	0.16

*Note:* Survival time was calculated since symptom onset (model entry at baseline visit). Data from 3833 patients from the ALS Center Ulm of whom 2214 died, and from 852 patients from the ALS Registry Swabia of whom 633 died. NIV, TIV, and PEG were analyzed in deceased patients only. Bold values indicate statistical significance.

Applying the multivariable Cox proportional hazards model (Table 3), in both datasets, age of onset (UC: HR 0.63 [95% CI: 0.56–0.73], *p* < 0.001, SR: HR 0.58 [95% CI: 0.48–0.69]; *p* < 0.001), spinal onset (UC: HR 0.67 [95% CI: 0.59–0.76], *p* < 0.001, SR: HR 0.82 [95% CI: 0.68–0.99]; *p* = 0.04), slower pre‐baseline progression rate (UC: HR 0.41 [95% CI: 0.36–0.47], *p* < 0.001, SR: HR 0.41 [95% CI: 0.34–0.49]; *p* < 0.001), higher BMI (UC: HR 0.85 [95% CI: 0.75–0.96], *p* < 0.001, SR: HR 0.82 [95% CI: 0.69–0.98]; *p* = 0.02), TIV (UC: HR 0.52 [95% CI: 0.36–0.75], *p* = 0.0005; SR: HR 0.19 [95% CI: 0.08–0.47]; *p* < 0.001), and PEG (UC: HR 0.80 [95% CI: 0.70–0.93]; *p* = 0.003, SR: HR 0.59 [95% CI: 0.46–0.75]; *p* < 0.001) were statistically significant. NIV (HR 0.76 [95% CI: 0.63–0.93], *p* = 0.01) was associated with longer survival in SR, but not in UC, while the effect of male sex was no longer significant in both cohorts. To consider the potential effect of both diverging time spans in both cohorts and the effect of the COVID‐19 pandemic, we also performed an additional analysis covering an identical time span between October 2010 and February 2020 in both cohorts (Table 4). This analysis, however, provided similar findings.

**TABLE 3:** Adjusted hazard ratios (HR) for different prognostic factors for survival with ALS.

	ALS Center Ulm (*N*_Deceased_ = 1012, *N*_Survived_ = 1043)			ALS Registry Swabia (*N*_Deceased_ = 529, *N*_Survived_ = 180)		
Exposures	*N*	HR (95% CI)	*p*	*N*	HR (95% CI)	*p*
Age of onset (< median)	2055	**0.63 (0.56, 0.73)**	**< 0.001**	709	**0.58 (0.48, 0.69)**	**< 0.001**
Sex (female vs. male)	2055	0.96 (0.85, 1.09)	0.56	709	1.09 (0.91, 1.30)	0.34
Spinal onset (vs bulbar onset)	2055	**0.67 (0.59, 0.76)**	**< 0.001**	709	**0.82 (0.68, 0.99)**	**0.04**
Pre‐baseline ALS‐FRS‐R progression rate (< median)	2055	**0.41 (0.36, 0.47)**	**< 0.001**	709	**0.41 (0.34, 0.49)**	**< 0.0001**
BMI at baseline (≥ median)	2055	**0.85 (0.75, 0.96)**	**0.009**	709	**0.82 (0.69, 0.98)**	**0.02**
NIV (ever vs. never)	1012	1.00 (0.88, 1.13)	0.97	529	**0.76 (0.63, 0.93)**	**0.009**
TIV (ever vs. never)	1012	**0.52 (0.36, 0.75)**	**< 0.001**	528	**0.19 (0.08, 0.47)**	**< 0.001**
PEG (ever vs. never)	1012	**0.80 (0.70, 0.93)**	**0.003**	529	**0.59 (0.46, 0.75)**	**< 0.001**

*Note:* Survival time was calculated since symptom onset (model entry at baseline visit). Age of onset (< median vs. ≥ median), sex, spinal onset (vs. bulbar onset), pre‐baseline ALSFRS‐R progression rate (< median vs. ≥ median), and BMI at baseline are mutually adjusted in 2055 patients from the ALS center Ulm of whom 1012 died, and in 709 patients from the ALS Registry Swabia of whom 529 died. NIV, TIV, and PEG are adjusted for age of onset (< median vs. ≥ median), sex, spinal onset (vs. bulbar onset), pre‐baseline ALSFRS‐R progression rate (< median vs. ≥ median), and BMI at baseline in 2055 patients from the ALS center Ulm of whom 1012 died, and in 709 patients from the ALS Registry Swabia of whom 529 died. Only deceased patients were considered for the analysis regarding NIV, TIV, and PEG. Bold values indicate statistical significance.

**TABLE 4:** Adjusted hazard ratios (HR) for different prognostic factors for survival with ALS (Oct 2010–Feb 2020).

	ALS Center Ulm (*N*_Deceased_ = 720, *N*_Survived_ = 830)			ALS Registry Swabia (*N*_Deceased_ = 527, *N*_Survived_ = 173)		
Exposures	*N*	HR (95% CI)	*N*	*N*	HR (95% CI)	*p*
Age of onset (< median)	1550	**0.65 (0.55, 0.76)**	**< 0.001**	700	**0.58 (0.49, 0.70)**	**< 0.001**
Sex (female vs. male)	1550	0.93 (0.80, 1.09)	0.37	700	1.09 (0.92, 1.30)	0.33
Spinal onset (vs bulbar onset)	1550	**0.76 (0.65, 0.89)**	**< 0.001**	700	**0.82 (0.68, 0.99)**	**0.04**
Pre‐baseline ALS‐FRS‐R progression rate (< median)	1550	**0.41 (0.35, 0.48)**	**< 0.001**	700	**0.41 (0.34, 0.49)**	**< 0.0001**
BMI at baseline (≥ median)	1550	0.88 (0.76, 1.02)	0.09	700	**0.83 (0.70, 0.99)**	**0.03**
NIV (ever vs. never)	720	1.02 (0.88, 1.20)	0.76	527	**0.77 (0.63, 0.94)**	**0.009**
TIV (ever vs. never)	720	**0.58 (0.37, 0.88)**	**0.012**	526	**0.19 (0.08, 0.46)**	**< 0.001**
PEG (ever vs. never)	720	**0.84 (0.70, 1.00)**	**0.046**	527	**0.58 (0.45, 0.74)**	**< 0.001**

*Note:* Survival time was calculated since symptom onset (model entry at baseline visit). Age of onset (< median vs. ≥ median), sex, spinal onset (vs. bulbar onset), pre‐baseline ALSFRS‐R progression rate (< median vs. ≥ median), and BMI at baseline are mutually adjusted in 1550 patients from the ALS center Ulm of whom 720 died, and in 700 patients from the ALS Registry Swabia of whom 527 died. NIV, TIV, and PEG are adjusted for age of onset (< median vs. ≥ median), sex, spinal onset (vs. bulbar onset), pre‐baseline ALSFRS‐R progression rate (< median vs. ≥ median), and BMI at baseline in 1550 patients from the ALS center Ulm of whom 720 died, and in 700 patients from the ALS Registry Swabia of whom 527 died. Only deceased patients were considered for the analysis regarding NIV, TIV, and PEG. Bold values indicate statistical significance.

## 4 Discussion

Our results revealed important differences regarding the demographic and clinical characteristics of the hospital‐ versus the population‐based cohort. Patients from the hospital cohort were younger (median 60.9 vs. 65.0 years) and had a higher share of male patients (60.5% vs. 56.3%), confirming previous studies [30, 31] and possibly indicating that these factors contribute to the willingness to travel larger distances to receive specialized treatment and/or to participate in clinical studies. The higher share of patients with a positive family history (12.9% vs. 6.3%), which also confirms previous reports [31], may likewise be explained by the fact that, along with the availability of genetic counseling and testing in a university setting, promising studies and treatments (including the ASO study for patients with SOD1 mutations; VALOR [38, 39] since 2016) were either available or planned for this subgroup.

Patients from the hospital‐derived database had a longer median diagnostic delay (10.5 vs. 6.9 months), which might be explained by the slower course of the disease as indicated by the lower pre‐baseline progression rate (−0.54 vs. −0.79 points/month). The slower progression rate, on the other hand, might explain the higher ALSFRS‐R at baseline (40.0 vs. 38.0).

A larger extent of disability implies that long travel distances are associated with significant burden, which might lower the patients’ willingness to visit a specialized center. Interestingly, the opposite constellation was observed when comparing the progression rates during follow‐up, that is between the first and the last recorded visit in each cohort (−0.78 vs. −0.53). This finding most likely reflects a selection bias, that is, patients featuring a rapid disease progression are more likely to seek advice from a tertiary ALS center. These findings are highly relevant for the conduct of clinical trials, as several recent publications have highlighted the need to address the heterogeneity of study populations, especially with regard to progression rates in ALS [29, 40, 41].

Differences between both data sets also extended to the frequency of NIV and PEG treatment, which were both more frequently performed in the hospital cohort (NIV: 34.3% vs. 25.3%, PEG: 26.7% vs. 17.7%). Besides the availability of both measures, other factors, such as how strongly NIV and PEG are recommended by the treating physician, may also contribute to this finding. This hypothesis is further corroborated by the fact that NIV and TIV rates significantly differ even among specialized centers within the same country, as a lower share of NIV (20.8% vs. 34.3%), but a higher share of TIV (9.5% vs. 2.6%) compared to Ulm was reported from the specialized ALS center Charité Berlin in a recent publication [14]. It is well known that the use of NIV and TIV is strongly influenced by practice and cultural differences [42]. In general, besides the personal preference and expertise of treating physicians, such inter‐center differences might also be influenced by infrastructural factors, for example, whether patients are preferably treated in an in‐ or out‐patient setting. Moreover, inconsistent study results might be influenced by varying timeframes within which the studies were conducted, as NIV techniques and practices have developed during recent years.

With regard to prognostic factors, there was an accordance between the hospital‐based cohort and the epidemiological registry regarding a positive impact of younger age of onset, spinal onset, higher BMI, and lower progression rates, which is in line with previous reports [4]. On the other hand, the positive effect of NIV, which has been established by a former randomized controlled trial [15], was only found in the registry, but not in the hospital‐based cohort, possibly indicating that complete longitudinal datasets over prolonged time periods are necessary to detect smaller effects, a lesson which has also been learned from recent clinical trials. For example, several clinical endpoints from the aforementioned VALOR study with the ASO tofersen were negative in the initial trial over 28 weeks [39], but positive when also considering the subsequent long‐term extension phase [38]. Another explanation might be that the hospital cohort comprises earlier timeframes, in which technical requirements of NIV (devices and masks) were less optimized and NIV might still have been underused.

Interestingly, on the other hand, a life‐prolonging effect of PEG was found in both cohorts applying the multivariable model, further supporting the view that PEG insertion is an effective, safe, and reliable measure in ALS. This is an important finding, as previous studies have provided heterogeneous results regarding the association between PEG insertion and survival [43, 44, 45].

In the Swabia registry, male sex was associated with longer survival in the univariate analysis, but not in the multivariable analysis, possibly indicating that this association might mainly rely on the fact that female patients more frequently feature a bulbar disease onset as a negative prognostic factor. In summary, our analysis of the effect of prognostic factors shows that [1] structural imbalances between study populations regarding demographic and clinical factors, [2] the length of observation periods, and [3] whether results are properly adjusted for confounders may at least partly explain seemingly inconsistent or even contradictory literature reports.

The present study is not without limitations as the analysis of observational data is prone to bias, and causal relationships are difficult to explore. In many cases, differences between both datasets can have a wide variety of reasons and are most likely multicausal. The hospital cohort covers a significantly larger time span compared to the registry cohort. Therefore, although we provided a sub‐cohort analysis for the overlapping time span, we cannot rule out that time‐dependent factors influenced the comparison. Moreover, it would have been informative to specifically analyze the subgroup of patients who were part of the registry but not part of the hospital cohort, which was, however, not possible due to the structure of the data.

However, keeping these limitations in mind, we believe that this study provides some valuable preliminary insights related to the difference between hospital‐ and population‐based data in two large cohorts originating from the same geographic region. These differences are most likely caused by a significant selection bias in the hospital‐based cohort, which implies potentially far‐reaching consequences concerning the interpretation of clinical trials. As study populations from clinical trials rely on specialized tertiary ALS centers, some study results might not be transferrable to the whole ALS population. On the other hand, potentially effective substances might be falsely rejected because the study population might not be representative. For example, recent studies have highlighted the importance of considering progression rates and indicated that a high share of slow progressors might potentially contribute to the negative outcome of a study [46, 47]. Here, we found that the hospital‐based cohort was biased toward slower progression rates at baseline, indicating the possibility that this factor might have unfavorably influenced the results of both studies. Therefore, further efforts seem necessary to enable all ALS patients to receive a specialized treatment and facilitate access to clinical studies.

## Author Contributions

**Johannes Dorst:** conceptualization, methodology, data curation, investigation, validation, formal analysis, supervision, visualization, project administration, writing – original draft, writing – review and editing. **Jens Dreyhaupt:** methodology, data curation, software, validation, visualization, formal analysis, writing – review and editing. **Deborah Wernecke:** methodology, software, data curation, validation, formal analysis, visualization, writing – review and editing. **Ulrike Weiland:** investigation, writing – review and editing. **Özlem Parlak:** investigation, writing – review and editing. **Maximilian Wiesenfarth:** investigation, writing – review and editing. **Zeynep Elmas:** investigation, writing – review and editing. **Christine Herrmann:** investigation, writing – review and editing. **Hansjörg Bäzner:** investigation, writing – review and editing. **Axel Boertlein:** investigation, writing – review and editing. **Silke Dempewolf:** investigation, writing – review and editing. **Christian Foerch:** investigation, writing – review and editing. **Martin Hecht:** investigation, writing – review and editing. **Andreas Kohler:** investigation, writing – review and editing. **Christian Opherk:** investigation, writing – review and editing. **Katharina Althaus:** investigation, writing – review and editing. **Monika Clauer‐Bredt:** investigation, writing – review and editing. **Alfred Lindner:** investigation, writing – review and editing. **Wolfgang Ruf:** investigation, writing – review and editing. **David Brenner:** investigation, writing – review and editing. **Simon Witzel:** investigation, writing – review and editing. **Raphael S. Peter:** methodology, software, data curation, validation, formal analysis, visualization, writing – review and editing. **Joachim Schuster:** methodology, writing – review and editing. **Albert C. Ludolph:** writing – review and editing, investigation. **Angela Rosenbohm:** methodology, writing – review and editing, investigation, conceptualization. **Gabriele Nagel:** conceptualization, methodology, software, data curation, validation, formal analysis, visualization, writing – review and editing, supervision.

## Conflicts of Interest

The authors declare no conflicts of interest.

## Data Availability

Individual participant data that underlie the results reported in this article, after de‐identification (text, tables, and figures) will be available. Data will be available beginning 3 months and ending 5 years following article publication. Data will be shared with researchers who provide a methodologically sound proposal. Data will be shared for analyses to achieve the aims in the approved proposal. Proposals should be directed to johannes.dorst@uni-ulm.de; to gain access, data requestors will need to sign a data access agreement.
